# Red Yeast Rice for the Improvement of Lipid Profiles in Mild-to-Moderate Hypercholesterolemia: A Narrative Review

**DOI:** 10.3390/nu15102288

**Published:** 2023-05-12

**Authors:** Arrigo F. G. Cicero, Federica Fogacci, Anca Pantea Stoian, Peter P. Toth

**Affiliations:** 1 Center for the Study of Hypertension and Related Cardiovascular Risk Factors, Medical and Surgery Sciences Department (DIMEC), University of Bologna, 40126 Bologna, Italy; 2 IRCCS AOU S. Orsola di Bologna, 40138 Bologna, Italy; 3 Department of Medicine and Surgery Sciences, University of Bologna, 40126 Bologna, Italy; 4 Department of Diabetes, Faculty of Medicine, Nutrition and Metabolic Diseases, Carol Davila University of Medicine and Pharmacy, 050474 Bucharest, Romania; 5 CGH Medical Center, Sterling, IL 61081, USA; 6 Division of Cardiology, Johns Hopkins University School of Medicine, Baltimore, MD 21205, USA

**Keywords:** efficacy, endothelial function, inflammatory markers, *Monascus purpureus*, safety, vascular remodeling

## Abstract

Reducing low-density lipoprotein cholesterol (LDL-C) levels is a key target for lowering cardiovascular risk and preventing atherosclerotic cardiovascular disease (ASCVD). Red yeast rice (RYR) is a nutraceutical widely used as a lipid-lowering dietary supplement. The main cholesterol-lowering components of RYR are monacolins, particularly monacolin K, which is structurally identical to lovastatin and targets the same key enzyme of cholesterol biosynthesis. RYR supplementation reduces LDL-C levels by approximately 15–34% versus placebo, with a similar effect to low-dose, first-generation statins in subjects with mild-to-moderate dyslipidemia. RYR has also demonstrated beneficial reductions of up to 45% versus placebo in the risk of ASCVD events in secondary prevention studies. RYR at a dose that provides about 3 mg/d of monacolin K is well tolerated, with an adverse event profile similar to that of low-dose statins. RYR is therefore a treatment option for lowering LDL-C levels and ASCVD risk for people with mild-to-moderate hypercholesterolemia who are ineligible for statin therapy, particularly those who are unable to implement lifestyle modifications, and also for people who are eligible for statin therapy but who are unwilling to take a pharmacologic therapy.

## 1. Introduction

Hypercholesterolemia, especially elevated low-density lipoprotein cholesterol (LDL-C), is a major modifiable risk factor for atherosclerotic cardiovascular disease (ASCVD) [1,2]. Guidelines recommend lifestyle changes and, when indicated, pharmacologic interventions, to lower LDL-C levels and decrease ASCVD risk [2,3,4]. However, some individuals with mild-to-moderate dyslipidemia and a low ASCVD risk who are not eligible for pharmacologic treatment may not achieve target LDL-C levels through lifestyle changes alone. These individuals, and also people who do not wish to take a pharmaceutical drug, might benefit from a nutraceutical such as RYR that helps them achieve lower LDL-C levels and lower their ASCVD risk.

The goal of treatment for hypercholesterolemia is to lower the burden of ASCVD and prevent ASCVD events such as myocardial infarction (MI), ischemic stroke, need for revascularization, and death [2]. Where pharmacologic intervention is recommended, statins form the mainstay of treatment [2,3,4]. Other lipid-lowering drugs such as ezetimibe, bempedoic acid, proprotein convertase subtilisin/kexin type 9 inhibitors, and inclisiran may be inaccessible to many people for a variety of reasons, such as high costs, lack of local regulatory approval, and/or restriction to higher-risk patient groups in treatment guidelines [2,3,4].

Red yeast rice (RYR) is a nutraceutical produced by fermentation of yeast, usually *Monascus purpureus*, in white rice [5]. The fermentation process produces numerous compounds, including pigments that cause the typical red coloration. Fermentation also produces monacolins, including the monacolin K subtype, which have lipid-lowering properties and are the principal active ingredients of RYR [5,6,7]. As a result, RYR has been used in Asia for centuries as a red food-coloring agent and medicinal food, and it is now widely used as a lipid-lowering dietary supplement [8]. However, the quality of RYR products can vary and they are treated differently by major regulators; products containing monacolin K are regulated as drugs in the United States [9], whereas in Europe they are treated as food supplements and are available without prescription [9].

Despite meta-analyses of randomized trials indicating that RYR reduces LDL-C levels versus placebo, with similar effects to low-dose, first-generation statins [10,11], international guidelines for the management of dyslipidemia and ASCVD prevention vary in their recommendations relating to RYR. The 2016 Joint Position Statement of the Italian Society of Diabetology and the Italian Society for the Study of Arteriosclerosis on nutraceuticals for the treatment of hypercholesterolemia states: “On the basis of current knowledge, the use of RYR preparations containing up to 10 mg/day monacolin K can be advised for patients with mild-to-moderate ASCVD risk and LDL-C levels exceeding the recommended therapeutic targets by up to 20–25% despite the implementation of adequate lifestyle changes” [12]. The 2019 European Society of Cardiology (ESC)/European Atherosclerosis Society (EAS) guidelines on the management of dyslipidemia state that nutraceuticals containing purified RYR can be considered in individuals with elevated plasma cholesterol who do not qualify for statins because of their global ASCVD risk [3], and the 2018 International Lipid Expert Panel provides a class I recommendation with level A evidence for the use of RYR in patients with statin intolerance [13,14]. Xuezhikang, a Chinese patent medicine with RYR as its primary component, is listed in the primary prevention guidelines for CVD in China [15]. However, the 2021 ESC guidelines on ASCVD prevention state that RYR supplements are not recommended [2], while the 2018 American College of Cardiology (ACC)/American Heart Association (AHA) guidelines on the management of blood cholesterol [4] and the 2019 ACC/AHA guidelines on the primary prevention of ASCVD [16] do not mention RYR.

This article aims to address the inconsistencies in the guidelines by critically reviewing clinical trial data, meta-analyses, and real-world surveillance on the metabolic effects and tolerability of RYR in lowering LDL-C and ASCVD risk. The main components isolated from RYR are listed in Table 1 [17]. 

## 2. Effect of RYR on LDL-C Plasma Level and Estimated Risk of ASCVD

### 2.1. Epidemiology and Natural History of Hypercholesterolemia and ASCVD

ASCVD is the leading cause of death globally, with ischemic heart disease being responsible for 16% of deaths [18], and it is a major cause of disability in developed countries [14]. A well-established, major, modifiable risk factor for the development of ASCVD is hypercholesterolemia, particularly elevated LDL-C [1,2], making lowering LDL-C levels a key target for reducing CVD risk [2].

The prevalence of hypercholesterolemia differs markedly by country. For example, in the United States, data from the National Health and Nutrition Examination Survey showed that the prevalence of hypercholesterolemia (total cholesterol [TC] ≥240 mg/dL) in adults aged ≥20 years was 11.4% during 2015–2018, with the greatest prevalence in adults aged 40–59 (15.7%), and no significant differences in hypercholesterolemia by race or Hispanic ethnicity [19]. For elevated LDL-C (≥130 mg/dL), the age-adjusted prevalence in the United States was 29.4% for 2015–2016 [20]. In contrast, in the Asia Pacific region, the prevalence of TC ≥240 mg/dL ranged from 9.0 to 44.9% across countries and survey years (2006–2015) [21], while in the United Kingdom, the crude prevalence of primary hypocholesterolemia and mixed dyslipidemia was estimated at 23.5% in 2019 [22]. Globally, the overall burden of high LDL-C levels is increasing when measured in terms of the total numbers of disability-adjusted life years (DALYs), deaths, years lived with disabilities, and years of life lost [23]. The total number of DALYs reached 98.6 million in 2019, with age-standardized DALY rates specifically due to high LDL-C highest in Eastern Europe, North Africa, the Middle East, Oceania, and Central Asia, and lowest in Australasia, Western Europe, Andean Latin America, and the high-income Asia Pacific [23].

### 2.2. Purpose and Outcome of Treatment

The management of LDL-C forms part of a comprehensive ASCVD risk-reduction strategy, which is based on individual CVD risk profiles and preferences [2,3]. Given that guideline recommendations for the management of dyslipidemia are based on ASCVD risk calculators, which are strongly dominated by age [2,3,4], many individuals do not reach the designated level of risk for intervention until middle age or older, leading to a delay in the initiation of therapy. However, by this time, such individuals may have been exposed to LDL-C levels for decades. 

Prolonged reduction in LDL-C levels is associated with a substantially decreased risk of ASCVD [1]. A meta-analysis of data from 170,000 participants in 26 clinical trials of lipid-lowering drugs demonstrated a reduction of approximately one-fifth in the risk of major ASCVD events per 1 mmol/L decrease in LDL-C in the secondary prevention setting, with a similar benefit in primary prevention [24]. Further meta-analyses have confirmed that the greater the absolute LDL-C reduction, the greater the ASCVD risk reduction [3]. Mendelian randomization studies indicate that, alongside absolute LDL-C level, the duration of exposure to higher LDL-C is significantly associated with ASCVD risk, demonstrating a cumulative impact of LDL-C throughout an individual’s lifetime [25,26]. One such study examined data from >300,000 participants and showed that the prolonged exposure to lower LDL-C mediated by nine polymorphisms in six different genes (i.e., beginning from early life) was associated with a three-fold greater risk reduction per unit lower LDL-C versus intervention with a statin started later in life [25]. Overall, this indicates a rationale for early and prolonged lipid-lowering interventions to achieve lifetime risk reduction.

The recommended LDL-C goals in the ESC/EAS guidelines for the management of dyslipidemia are based on the patient’s ASCVD risk, with goals of <116 mg/dL (<3.0 mmol/L) and <100 mg/dL (<2.6 mmol/L) for patients at low and moderate risk, respectively [3]. Tighter control of LDL-C is recommended for patients at high and very high risk, with targets of a ≥50% reduction in LDL-C from baseline and an absolute LDL-C <70 mg/dL (<1.8 mol/L) for high risk and <55 mg/dL (<1.4 mmol/L), or even <40 mg/dL (<1.0 mmol/L), in selected patients for very high risk [3]. 

Dietary modifications—avoiding trans fats, lowering intake of saturated fats and cholesterol, and increasing fiber intake—all lower LDL-C by levels 5–10% [3]. Additional lowering of LDL-C by nutraceuticals such as RYR might help people meet their LDL-C goals, particularly individuals with elevated LDL-C levels who do not qualify for treatment with statins because they have a low global CV risk [3] and those who are unwilling to use statins [27]. Other lipid-lowering nutraceuticals are beyond the scope of this review, but a selection of the preparations available is shown in Table 2.

### 2.3. Metabolism of the Bioactive Components and Mechanism of Action of RYR

**Figure 1:**
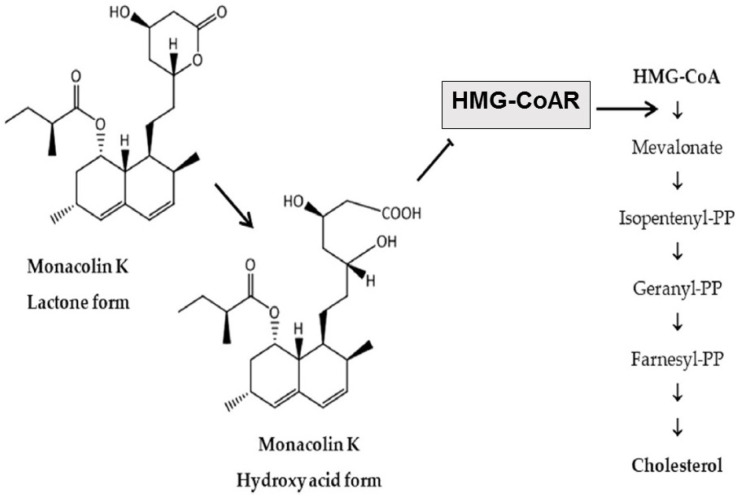
Main cholesterol-lowering mechanism of action of RYR [28]. RYR = red yeast rice. HMG-CoA = β-hydroxy β-methylglutaryl coenzyme A; HMG-CoAR = β-hydroxy β-methylglutaryl coenzyme A reductase; PP = pyrophosphate.

**Figure 2:**
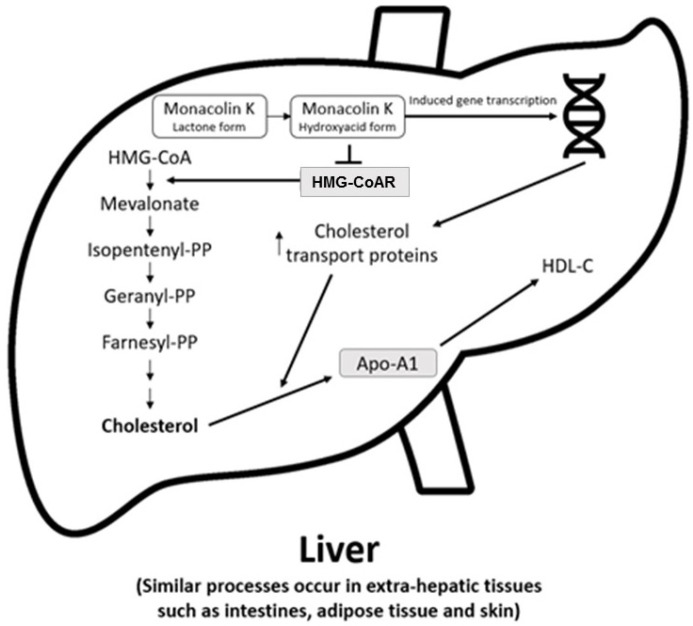
Actions of RYR on cholesterol synthesis, transport, and gene expression [35]. Apo-A1 = apolipoprotein A1; HDL-C = high-density lipoprotein cholesterol; HMG-CoA = β-hydroxy β-methylglutaryl coenzyme A; HMG-CoAR = β-hydroxy β-methylglutaryl coenzyme A reductase; PP = pyrophosphate.

The main cholesterol-lowering effects of RYR are provided by monacolins, its main bioactive components. Monacolin K, the most abundant monacolin in RYR [28], occurs in two forms: the lactone (inactive) and hydroxyl acid (active) [28,29,30]. The proportion of the acid form has been reported to vary from 5% to 100%, depending on pH: at low pH, the lactone form predominates, whereas at neutral and acidic pH, the acid form predominates [5,28,29]. The lactone form of monacolin K is structurally identical to lovastatin [28,29]; therefore, similar to other statins, monacolin K is a reversible inhibitor of β-hydroxyβ-methylglutaryl coenzyme A reductase (HMG-CoAR), the enzyme catalyzing the rate-limiting step of cholesterol biosynthesis (Figure 1) [5]. Lovastatin is a prodrug that must be hydrolyzed to the acid form [5,28,29] because only this form bonds to key amino acid residues in the binding pocket of HMG-CoAR (differing from those bound by the lactone form), stabilizing the interaction between the two molecules [31]. By contrast, the acid form is naturally present in RYR, and this can result in differences in bioavailability and clinical profiles between RYR and lovastatin. In addition to inhibition of HMG-CoAR, statins have demonstrated actions at the transcription level, with increases in nuclear receptor peroxisome proliferator-activated receptor alpha (PPARα), mediated by the Rho-signaling pathway, potentially explaining increases in high-density lipoprotein cholesterol (HDL-C) levels with statin therapy [32]. Statins also influence the expression of a wide range of other genes, including transcription factors involved in inflammation, proliferation, and differentiation [33,34], and cholesterol transporters in a range of tissues, including the liver, intestine, adipose tissue, and skin, and apolipoprotein A1 (apo-A1) in the liver (Figure 2) [35]. This leads to an increased transfer of cholesterol to apo-A1, leading to a greater production of HDL. 

Similarly to lovastatin, CYP3A4 is involved in the metabolism of monacolins which are substrates of P-glycoprotein. Their bioavailability and safety could therefore be affected by interactions with other medicinal products and foods, although these potential effects are yet to be fully characterized [29,36]. Plasma concentrations of the hydroxy acid and lactone forms of monacolin K after ingestion of 2400 mg RYR are much lower than those of the corresponding forms of lovastatin after taking a 20 mg conventional tablet [37]; this is expected to reduce the risk of adverse effects.

RYR supplements differ widely in their monacolin K content, resulting in a daily dose range of approximately 0.1–14.5 mg [38,39,40]. This is partly because the relative abundances of monacolin K and other monacolin subtypes in RYR, including monacolins J, L, and X, are affected by the fermentation conditions and yeast strain used during their production [30,39,40]. In addition, RYR contains non-monacolin components, including sugars (25–73% mainly starch), proteins (14–31%), water (2–7%), fatty acids (1–5%), and other bioactive components such as pigments, sterols, isoflavones, and citrinin (discussed below) [5,17]. The contributions of components other than monacolins to the cholesterol-lowering properties of RYR supplements are difficult to assess due to their varying compositions and very small concentration.

A study in healthy volunteers taking an RYR product reported that the pharmacokinetic properties of both forms of monacolin K (lovastatin and lovastatin acid) were linear across the range of 1–4 capsules taken as a single dose, with no significant accumulation after multiple dosing [36].

Asian ethnicity is associated with a higher statin exposure than in non-Asians at a given dose, partly due to genetically determined differences in transport mechanisms [41]. The approved maximum doses of most statins are therefore lower in some Asian countries (e.g., Japan) than elsewhere [41]. The greater exposure in Asians than Caucasians is more marked for some statins (e.g., rosuvastatin) [42] than for others (e.g., atorvastatin) [43]. 

### 2.4. Effects of RYR on Lipids

#### 2.4.1. RYR versus Placebo

Randomized trials have investigated a variety of RYR and monacolin K daily doses versus placebo for primary and secondary CVD prevention in subjects with dyslipidemia (Table 3) [44,45,46,47,48,49,50,51,52,53,54,55]. Other components of the RYR formulations, where described, are shown in Supplemental Table S1. RYR treatment of at least 4 weeks’ duration demonstrated significant reductions in LDL-C of approximately 15–34% from baseline (*p* < 0.05); significant differences versus placebo were also observed. In several of these studies, the daily dose of monacolin K (where stated) was less than 10 mg/day [44,48,49,53,54,55], below the 10–80 mg/day recommended for the structural analog lovastatin. Significant decreases were also seen for TC compared with baseline and/or placebo [44,45,46,47,48,49,50,51,52,53,54,55], while effects on HDL-C and triglycerides were generally small and inconsistent [44,45,46,47,48,49,50,51,52,53,54,55] (Supplemental Table S2). Several of these studies also reported significant decreases in apolipoprotein B levels from baseline with RYR (−27% to −19%; all *p* < 0.001) [45,46,47,49,52,55], and small, mostly non-significant increases in apo-A1 [45,46,47,49,52] (Supplemental Table S2).

#### 2.4.2. RYR versus Other Statin Preparations

Clinical trials comparing RYR with other statin preparations [56,57,58,59,60,61,62,63,64,65,66] have reported reductions in LDL-C from baseline of up to 33.4%, generally similar to the statin comparator in each study. However, most of these studies were performed in China and are not of high quality. In addition, genetically determined differences in transport mechanisms in East Asians mean that RYR seems to be more effective in Chinese people [41], and this might contribute to the impressive results versus other statins. The finding that RYR, even at a low dose, has the same effect as high-intensity statins, is therefore not reliable. These trials included two studies with a formulation (Armolipid Plus^®^, Meda-Mylan, Monza, Italy) providing monacolin K 3 mg/day [59,60]. This formulation is discussed further in Section 2.7. Certain studies have also reported that RYR significantly reduced TC [59,60,61,62,63,65,66] and triglycerides [56,57,58,62,65,66], while effects on HDL-C were generally small and inconsistent [57,58,59,60,62,63,64,65,66] (Supplemental Table S2). 

#### 2.4.3. RYR versus Placebo and Other Statins Meta-Analyses

A 2021 meta-analysis of 15 high-quality randomized controlled trials (mostly using doses of 600–2400 mg twice daily) involving 1012 subjects showed that RYR significantly decreased LDL-C versus placebo (mean difference [MD] −35.82 mg/dL; 95% confidence interval [CI] −43.36, −28.29 [*p* < 0.00001]), with no significant difference versus other statins (simvastatin 20 mg/d, pravastatin 40 mg/d, or rosuvastatin 10 mg/d) with an MD of 1.89 mg/dL (95% CI −7.93, 11.71 [*p* = 0.71]) [11]. RYR significantly increased HDL-C versus placebo (MD 3.47 mg/dL; 95% CI 0.94, 6.00 [*p* = 0.007]) to a similar degree as other statins (MD 2.50 mg/dL; 95% CI −4.21, 9.22 [*p* = 0.46]). While RYR significantly reduced TC versus placebo (MD −37.43 mg/dL; 95% CI −47.08, −27.79 [*p* < 0.00001]), it was less effective than other statins (MD 12.24 mg/dL; 95% CI 2.19, 22.29 [*p* = 0.02]). For triglycerides, significant decreases were seen for RYR versus placebo (MD −20.65 mg/dL; 95% CI −35.60, −5.70 [*p* = 0.007]) and versus other statins (MD −19.90 mg/dL; 95% CI −32.22, −7.58 [*p* = 0.002]) [11]. 

An earlier meta-analysis of 20 randomized trials involving 6663 subjects showed that RYR (1200–4800 mg/d) was more effective than a placebo at reducing LDL-C (MD −1.02 mmol/L; 95% CI −1.20, −0.83 [*p* < 0.00001]) and TC (MD −1.00 mmol/L; 95% CI −1.23, −0.77 [*p* < 0.00001]) [10]. The effect of RYR was similar to that of low-intensity/low-dose statins on LDL-C (MD 0.03 mmol/L; 95% CI −0.36, 0.41 [*p* = 0.89]) and TC (MD −0.05 mmol/L; 95% CI −0.28, 0.18 [*p* = 0.67]) [10]. Finally, a network meta-analysis of 47 randomized controlled trials involving 4824 subjects, evaluating three different Chinese RYR supplements (most commonly Xuezhikang 600 mg twice daily), showed that RYR significantly reduced LDL-C, TC, and triglycerides versus placebo [67]. No significant differences in the levels of these parameters were observed with RYR versus simvastatin, although a ranking analysis suggested that Xuezhikang (surface under the cumulative ranking [SUCRA] value 82.6%) was more likely than simvastatin (SUCRA value 74.9%) to be effective in reducing LDL-C [67].

### 2.5. Effects of RYR on Inflammatory and Vascular Remodeling Biomarkers and Endothelial Function

Statins have been shown to reduce levels of the inflammatory biomarker high-sensitivity C-reactive protein (hs-CRP) [68], and this is also true of RYR, with reductions in hs-CRP versus placebo observed in subjects with mild hypercholesterolemia (−23.77%, 95% CI −30.54, −17.01) [50] and in those with coronary heart disease (CHD) (−50.0% versus −25.4%, *p* < 0.05) [46]. Significantly greater reductions in hs-CRP have also been reported for RYR versus simvastatin (*p* < 0.05) in subjects with unstable angina pectoris and statin-induced elevated liver enzymes [58]. 

Additionally, compared with a placebo, RYR treatment resulted in more favorable decreases of −28.05% (95% CI −35.18, −20.93) and −27.19% (95% CI −36.21, −18.15) in the vascular remodeling biomarkers matrix metalloproteinase (MMP)-2 and MMP-9, respectively, in subjects with mild hypercholesterolemia [50]. Moreover, 6 weeks’ treatment with RYR (Xuezhikang, 1200 mg/d) has been reported to significantly improve endothelial function (*p* < 0.05) measured by both pre-prandial and post-prandial flow-mediated vasodilation in subjects with CHD, while no change was seen with a placebo [46].

### 2.6. Beneficial Effects of Exposure to RYR on ASCVD Risk and Events

#### 2.6.1. RYR versus Placebo

The effects of RYR compared with placebo on ASCVD risk and events have been evaluated in clinical studies and meta-analyses in varying frail subject populations with a history of MI and/or CHD (Table 4). 

In a randomized Chinese study of almost 5000 subjects with a previous MI and average LDL-C levels at baseline, those taking RYR (Xuezhikang) daily for a mean of 4.5 years experienced a lower relative risk of non-fatal MI and death from CHD, ASCVD mortality, and total mortality by 45%, 30%, and 33%, respectively, compared with those taking a placebo [69]. Similar reductions in ASCVD events versus placebo were observed in subgroups from this trial of hypertensive subjects [70] and elderly (aged ≥ 65 years) hypertensive subjects treated with RYR [71].

Meta-analyses in subjects with a history of MI and/or CHD, including those with metabolic syndrome (diabetes or hypertension plus dyslipidemia) showed that, compared with subjects taking a placebo, those taking RYR had a lower risk of ASCVD endpoints including non-fatal MI (risk ratio (RR) 0.42, 95% CI 0.34, 0.52), revascularization (RR 0.58, 95% CI 0.48, 0.71), and sudden death (RR 0.71, 95% CI 0.53, 0.94) [72], as well as major ASCVD events (RR 0.54, 95% CI 0.43, 0.66) and mortality (RR 0.62, 95% CI 0.49, 0.78) [73] (Table 4).

#### 2.6.2. RYR versus Other Statins

A real-world, retrospective, population-based cohort study of Taiwan’s National Health Insurance Program in individuals without a history of stroke reported a lower risk of stroke in subjects taking RYR than age- and sex-matched controls who received the monacolin K analog lovastatin (hazard ratio [HR] 0.65, 95% CI 0.59, 0.71) [74]. However, hypertension and diabetes were significantly more common in the lovastatin cohort at baseline, which may have increased the baseline risk of stroke in the lovastatin versus RYR cohort [74]. 

In a comparison of RYR and simvastatin in 90 Chinese subjects with unstable angina pectoris and elevated liver enzymes associated with simvastatin, a significantly lower proportion of subjects receiving RYR or who continued taking simvastatin experienced ASCVD endpoints versus those who stopped taking simvastatin [58].

### 2.7. Beneficial Effects of RYR-Berberine Combinations

In order to lower the dose of RYR and improve tolerability while potentially increasing the cholesterol-lowering effect, combinations of RYR with other nutraceutical compounds that have different lipid-lowering mechanisms of action have been investigated.

One of the most extensively studied RYR nutraceutical combinations (Armolipid Plus^®^) includes berberine, a plant alkaloid that enhances the hepatic uptake of cholesterol, for which the largest pharmacovigilance datasets are available [75,76,77]. Armolipid Plus^®^ contains 3 mg monacolin K, 500 mg berberine, and 10 mg policosanols (aliphatic alcohols derived from sugar cane, reported to have cholesterol-lowering effects [78]). A meta-analysis of 12 randomized control trials involving 1050 subjects with up to 12 months’ follow-up showed that this preparation significantly decreased body-mass index (MD −0.25 kg/m^2^; *p* = 0.008), LDL-C (MD −26.67 mg/dL; *p* < 0.001), TC (MD −25.07 mg/dL; *p* < 0.001), triglycerides (MD −11.47 mg/dL; *p* < 0.001), fasting glucose (MD −3.52 mg/dL; *p* < 0.001), and hs-CRP versus placebo (−0.61 mg/L; *p* = 0.022), and significantly increased HDL-C (MD 1.84 mg/dL; *p* < 0.001) versus placebo [76]. For further details of these trials please see the meta-analysis publication [76].

Other RYR combinations with antioxidants, including coenzyme Q10, have also demonstrated lipid-lowering effects and decreases in hs-CRP compared with placebo [79,80]. In a crossover study in 25 subjects with moderate hypercholesterolemia, 4 weeks’ treatment with RYR containing 10 mg monacolins combined with antioxidants showed more favorable percentage changes from baseline than a placebo in TC (−18.35% versus −5.39%), LDL-C (−22.36% versus −1.38%), non-HDL-C (−22.83% versus −7.15%), and hs-CRP (−2.33% versus 2.11%) [79].

In a separate study in 40 subjects with moderate hypercholesterolemia, 6 months’ treatment with RYR containing 10 mg monacolins plus 30 mg coenzyme Q10 significantly reduced LDL-C versus placebo (−26.3% versus +3.4%, *p* < 0.05) [80]. Such combinations also demonstrated improvements compared with placebo in endothelial function assessed via pulse volume displacement (*p* < 0.05) and arterial stiffness assessed via pulse wave velocity (*p* < 0.05) in subjects with moderate hypercholesterolemia [79]. The potential benefits of these lipid-lowering and vascular remodeling effects from RYR combinations on CV risks and outcomes remain to be elucidated [76]. 

### 2.8. Convenience and Preference, and Health Economic Impact

Specific studies relating to the cost-effectiveness, convenience, and patient preference for RYR have not been identified. However, depending on the country, high-quality, highly purified RYR products could cost more than generic statins [5]. Additionally, while lipid-lowering drugs may be reimbursed by healthcare systems, the cost of RYR supplements is likely to fall on consumers unless they are classified as medicines, and this may limit their use [9]. Conversely, economic inequalities and geographic location markedly affect the proportion of people who take ASCVD medications, including lipid-lowering drugs [81,82]. For example, one study reported that statin use for secondary prevention was 66.5% in high-income countries compared with only 3.3% in low-income countries [82]. Statin use was also higher in urban versus rural areas within countries (19.9% versus 12% overall; *p* < 0.0001), with the greatest variation in the lowest-income countries [82]. Thus, RYR may be more easily accessible than pharmacologic interventions in some lower- or middle-income countries.

An additional aspect contributing to preference for RYR is that many individuals experience a real or perceived intolerance to statins [9], often relating to toxicity concerns [83]. Indeed, a key factor related to non-adherence to lipid-lowering drugs in patients with dyslipidemia is presenting with adverse events (AEs) or expressing concerns about them [84]. Adherence to lipid-lowering treatment is also associated with cost, with treatment persistence over 2 years found to be greater for nutraceuticals than for statins in 628 subjects with moderate hypercholesterolemia (odds ratio 1.29, 95% CI 1.14, 1.38) who were fully responsible for paying for treatment [85].

## 3. Safety and Tolerability of RYR

A large number of trials of RYR has been carried out on generally healthy mildly hypercholesterolemic subjects, without any evidence of toxicity [86]. The lactone form of monacolin K is structurally identical to lovastatin; it might therefore be expected to produce similar adverse effects to statins. However, numerous studies have shown that RYR is generally well tolerated [5,14]. Statin-related AEs, including AEs leading to discontinuation of therapy, creatine kinase elevation, elevation of liver function tests, and muscle symptoms, have been reported to be dose dependent [87], although the evidence for this relationship in primary prevention is limited [88]. It may therefore be expected that RYR formulations containing a low daily dose of monacolins would be better tolerated than pharmaceutical statins at high doses. However, the lack of standardization and quality control for the preparation of dietary supplements such as RYR, for example in the European Union and the United States, means that the amount of monacolin K and other constituents in RYR supplements, including the mycotoxin by-product of the fermentation process, citrinin, can vary widely [5,89]. In animals, citrinin has nephrotoxic, embryotoxic, and teratogenic effects [89,90], whereas in humans taking RYR, citrinin-related AEs have not been reported. The European Food Safety Authority has determined that citrinin intake should not exceed 0.2 µg/kg body weight/d [91], and good-quality RYR products on the market are certified citrinin free [5,89]. These issues of standardization are common among dietary supplements, with the potential for varying concentrations of active ingredients, substitution of less effective (or ineffective) substances, contamination with pathogens, or the presence of toxic substances [92]. The use of high-quality, purified RYR nutraceuticals produced under good manufacturing practice (GMP) conditions is therefore essential.

### 3.1. Clinical Trials

Overall, RYR has been well tolerated in clinical trials with 4–24 weeks of treatment in diverse populations with dyslipidemia, including those who are intolerant to statins [44,47,49,51,52,53,54,55,93]. AEs reported in randomized controlled trials have included musculoskeletal, gastrointestinal, hepatic, and general AEs, such as fatigue and dizziness, and laboratory observations such as elevated transaminases, which are rarely serious (Table 5) [44,47,48,49,51,52,54,55,57,60,63,64,66]. Many of these studies, including those with the greatest numbers of participants, have been conducted in China, and these have shown no increase in the incidence of AEs compared with control groups [73,86]. Despite the higher statin exposure observed for Asian versus non-Asian patients, a large cohort study reported no greater risk of serious AEs in Chinese than in non-Chinese older adults with similar indicators of general health [94].

### 3.2. Meta-Analyses

The low rates of AEs in clinical trials of RYR, as well as short-term follow-up, limited sample sizes, and incomplete reporting of safety in some studies, make meta-analyses of high-quality trials an invaluable source of RYR safety data. Five meta-analyses, including 53, 20, 12, 15, and 22 studies, respectively, concluded that RYR supplements were generally well tolerated, with similar rates of AEs, including musculoskeletal disorders, liver abnormalities, and kidney injury, and serious AEs, as control groups (placebo or other statin) [10,11,76,86,95] (Supplemental Table S3). One of these studies was the meta-analysis of Armolipid Plus^®^ described above [76]. It concluded that this formulation did not increase the risk of musculoskeletal or gastrointestinal disorders, nor increase aspartate aminotransferase or creatine phosphokinase levels, but was associated with a slight but clinically insignificant increase in alanine aminotransferase compared with placebo [76]. 

### 3.3. Real-World Evidence

Real-world reports from national surveillance systems provide important information on the safety of RYR-containing supplements, including combinations (Supplemental Table S4). 

The Italian Surveillance System of Natural Health Products collected 52 reports containing 55 suspected AEs to RYR-containing dietary supplements [96]. In all except 4 of these reports, the daily dose of monacolin K of the RYR supplements involved was 3 mg. Musculoskeletal and connective tissue (20 reactions [36%]), gastrointestinal (12 [22%]), hepatobiliary (10 [18%]), and skin and subcutaneous tissue disorders (9 [16%]) were the most frequently reported system organ classes (SOCs) for AEs. In 14 reports (27%), the AE was serious, with 13 (25%) requiring hospitalization, notably including 6 of 10 hepatic AEs. One case of rhabdomyolysis was considered certain to be caused by the RYR supplement, while 56% of all reactions were considered probably associated with RYR. The authors concluded that the safety profile of RYR is similar to that of other statins.

A total of 94 reports of 187 suspected AEs to RYR were collected by the Netherlands Pharmacovigilance Centre Lareb [97]. Of the 94 reports, 55 (59%) also involved concomitant medication use. The most frequently reported SOCs were musculoskeletal and connective tissue (64 reactions [34%]) and gastrointestinal disorders (33 [18%]), followed by general (23 [12%]) and nervous system disorders (16 [9%]). Six cases were classified as serious, of which three were acute pancreatitis, two cases had symptoms of rhabdomyolysis, and one case experienced acute hepatic failure. Overall, the role of RYR was considered as certain in two cases: one case of myalgia and one case of abdominal pain.

In 2018, the European Food Safety Authority reviewed the safety data for monacolin K in RYR from four sources (World Health Organization [2002–2018], the French Phytovigilance surveillance system [2009–2013], the Italian surveillance system [2002–2015], and the US Food and Drug Administration [2004–2017]) [29]. In total, 328 reports of AEs from RYR were reported across these four sources. Similar AE profiles were seen across these sources as those collected by the individual Italian and Dutch systems, with musculoskeletal and connective tissue AEs (~30–37% of cases), gastrointestinal AEs (12–23% of cases), skin and subcutaneous AEs (8–17% of cases), and hepatobiliary system AEs (9–32% of cases) being the most common. Rhabdomyolysis was rare, with four events reported across the French, Italian, and US systems. 

Of the three reports described above, only the Italian survey describes data on rechallenge, and in only seven subjects [96]. In the absence of rechallenge, the association between RYR and the events reported is unproven.

An additional source of RYR safety data is a post-marketing product-based database for Armolipid^®^/Armolipid Plus^®^ that has collected 542 case reports with 855 AEs [75]. For these RYR products, the daily dose of monacolin K was 3 mg. Based on an estimated 2,287,449 exposed consumers, the rate of AEs was 0.037%, with 26 suspected serious AEs in 21/542 cases (0.0009% of exposed consumers). There are no data on rechallenges.

A real-world retrospective cohort study of Taiwan’s National Health Insurance records suggested that RYR use was associated with a decreased risk of incident diabetes versus lovastatin in age- and sex-matched cohorts, with an HR of 0.46 (95% CI 0.43, 0.50) [98]. 

## 4. Discussion

New risk thresholds for pharmacologic treatment in the 2021 ESC guidelines on ASCVD prevention [2] are reducing the eligibility for statins of apparently healthy individuals with mild-to-moderate hypercholesterolemia [99]. Estimating the effect of RYR on the risk of ASCVD events in a primary prevention population, who are generally relatively young and healthy, may be misleading with current risk calculators, which are dominated by age and focus on 10-year risk. Instead, the rationale for using RYR is to provide a reduction in LDL-C that, although numerically modest, may be achieved for decades with habitual use. This has the potential to reduce the lifetime ASCVD risk [25]. In a recent network meta-analysis [100], RYR demonstrated a positive effect on LDL-C compared with commonly used lipid-lowering nutraceuticals (for instance phytosterols). RYR also has the advantage that it can be consumed once daily compared with other natural compounds such as artichoke extracts or bergamot polyphenols.

RYR provides lipid-lowering effects similar to those of low-dose, low-intensity statins [10,11] in subjects with mild-to-moderate hypercholesterolemia, with clinical-trial and real-world data demonstrating a favorable safety and tolerability profile similar to that of other statins [10,11,75,96]. Significant reductions of LDL-C have been reported with formulations containing doses of monacolin K as low as 2–3 mg/day [53,55,59,60], at which the risk of AEs can be expected to be low. Other AEs have been reported for RYR supplements containing higher doses of monacolin K than the supplements usually approved for use in Europe and elsewhere, and also for supplements with unknown RYR quality where the lack of rechallenge casts doubt on the cause–event relationship.

Clinical trials have shown that RYR is effective and well tolerated in patients intolerant to statins [48,58,60,93], and the International Lipid Expert Panel provides a class IA recommendation for RYR in this setting [13,14]. In patients with an indication for statin therapy, it is important to continue treatment because discontinuation is associated with an increase in cardiovascular events [83]. Strategies for resumption or continuation of therapy include reduction in the dose (or dose frequency, e.g., alternate-day dosing) or switching to a different statin [83,101,102]. The use of RYR containing a low dose of monacolin K could be a logical alternative to a conventional statin for such patients. RYR, being a food supplement, may be less vulnerable to the “nocebo” effect—a perception of AEs resulting from an expectation of harm—than a pharmaceutical preparation. 

## 5. Conclusions

RYR is an option for lowering LDL-C levels in individuals with mild-to-moderate hypercholesterolemia who are not eligible for pharmacologic treatment, particularly those who are unable to implement lifestyle modifications, and also for individuals who are intolerant to statins or who prefer to take a nutraceutical rather than a pharmacologic product. Given the large number of RYR nutraceutical products available and the limited regulatory oversight in some countries, physicians and recipients should choose a high-quality, purified RYR nutraceutical with a suitably low dose of monacolin K, produced under good manufacturing practice (GMP) conditions that is certified citrinin free. This will ensure consistent monacolin K dosing, help to reduce the risk of side effects, and minimize potential AEs due to contaminants. In the future, further studies will help to define the place of RYR in the management of dyslipidemia. For example, the role of components of RYR other than monacolins in lipid-lowering effects and their interactions with monacolin K, require further investigation, and a trial comparing an RYR preparation with a pharmaceutical containing the same dose of lovastatin could be informative [103]. Combinations of other nutraceuticals with RYR also require further clinical trials to establish the risk–benefit ratios and cost-effectiveness of these options. For patients with mild hypercholesterolemia who cannot tolerate statins and related products, ranges of alternative natural products are being investigated. Compounds derived from *Protium heptaphyllum* gum resin, belonging mainly to the ursane, oleanane, and tirucallane groups, have been shown to reduce cholesterol production and regulate the expression of proteins involved in their metabolism in human hepatocytes [31]. Herbs including basil, thyme, and sage contain lutein, zeaxanthin, vitamin E, and various polyphenols and have antioxidant activity [104]. Fruits including pomegranate and apricot contain various ingredients, including phenolic compounds, anthocyanins, and vitamins A, C, and E, with antioxidant and free radical scavenging actions, which may be more potent when they act in combination [105,106]. Omega-3 fatty acids are abundant in fish oils and marine microorganisms, peptides extracted from fish have cholesterol-reducing actions, and carotenoids and phenolic compounds with antioxidant activity have been derived from crustaceans and seaweeds [107]. The current evidence, however, shows that RYR delivers a modestly effective dose of monacolin K, and has other components that may add to the benefits of treatment.

## Figures and Tables

**Table 1:** Main components isolated from red yeast rice [17].

Component	Number Identified
Monacolins (including monacolins K, L, Q, R, and S)	23
Pigments	25
Organic acids and amino acids	9 (including citrinin)
Sterols	9
Decalin derivatives	7
Flavonoids	2
Terpenoids	5
Lignans	2
Coumarin	1
Polysaccharides	9

**Table 2:** Other nutraceuticals with lipid-lowering properties [6,14].

Name	Mechanisms of Action	Main Lipid-Lowering Component(s)	Effects on Lipids	Safety and Tolerability
Artichoke leaf extract	Inhibition of liver cholesterol synthesis via action on HMG-CoAR; effects on sterol regulatory element binding protein and acyl-CoA acyl transferase (ACAT)	Luteolin	Up to 10% reduction in LDL-C; small reduction in TG	Transient minor GI effects
Bergamot	Inhibition of liver cholesterol synthesis via inhibition of HMG-CoAR and ACAT; may also increase fecal cholesterol excretion and reduce intestinal cholesterol absorption of bile acids	Brutieridin, melitidin, neoeriocitrin	Up to 15% reduction in LDL-C; small reduction in TG	
Rice bran oil	Inhibition of liver cholesterol synthesis via inhibition of HMG-CoAR; reduction in intestinal cholesterol absorption	Gamma-oryzanol	0.18 mmol/L (7 mg/dL) reduction in LDL-C across 11 RCTs (*p* < 0.001)	No known side effects
Garlic	Inhibition of liver cholesterol synthesis via inhibition of HMG-CoAR, squalene mono-oxygenase, and acetyl-CoA synthetase; may also promote bile acid excretion	Allicin	Up to 5% reduction in LDL-C	Minimal, mostly GI side effects
Green tea extracts	Antioxidant effects; may also interfere with cholesterol absorption and inhibitcholesterol synthesis via inhibition of HMG-CoAR	Catechins, including epigallocatechin-3-gallate	Up to 5% reduction in LDL-C	Potential iron and folate deficiency with high doses; rare GI side effects, rash, transient elevation of BP
Vitamin B5 derivatives	Inhibition of fatty acid and cholesterol synthesis	Pantethine	Up to 11% reduction in LDL-C; smaller reductions in TG and total cholesterol	Well tolerated
Omega-3	Reduced VLDL and TG synthesis; increased fatty acid oxidation	Docosahexaenoic acid; eicosapentaenoic acid	25–30% reduction in TG levels; variable effects on LDL-C depending on components	Well tolerated, rare abdominal discomfort; fishy aftertaste
*Coptis*, *Hydrastis*, and *Berberis* spp.	Increased LDL-C excretion via increased expression of hepatic LDL receptors via inhibition of PCSK9	Berberine	~15% reduction in LDL-C; smallreduction in TG	Mild-to-moderate GI effects
Lupin	Unclear; multiple proposed mechanisms	Bioactive peptides; isoflavones	12% reduction in LDL-C; increase in HDL-C in some studies	Well tolerated; minor GI events
Soy	Unclear; multiple proposed mechanisms	Bioactive peptides; isoflavones	Up to 5% reduction in LDL-C; small reduction in TG and increase in HDL-C	Long-term use of high doses may disrupt fertility and thyroid function; may reduce absorption of calcium and other minerals
Turmeric	Unclear; multiple proposed mechanisms	Curcumin	Inconsistent effects reported; some studies report significant improvements in LDL-C, TG, and HDL-C	Well tolerated

BP = blood pressure; GI = gastrointestinal; HDL-C = high-density lipoprotein cholesterol; LDL-C = low-density lipoprotein cholesterol; PCSK9 = proprotein convertase subtilistin/kexin type 9; RCTs = randomized controlled trials; TG = triglycerides; VLDL = very low-density lipoprotein.

**Table 3:** Designs and LDL-C levels/changes in key randomized controlled trials of RYR versus placebo or other statins in subjects with dyslipidemia.

Study	Population	Primary/Secondary Prevention	Interventions	Study
**Placebo comparator**				
Heber, 1999 [44]	Dyslipidemia *n* = 88	Primary	RYR 2400 mg/d (MK 4.8 mg/d) vs. PBO 12 weeks	CFB Wk 12: RYR −1.01; PBO −0.13 *p* < 0.001 vs. PBO for LDL-C concentrations at Wk 12
Zhao, 2003 [45] Zhao, 2004 [46]	CHD *n* = 50	Secondary	XZK 600 mg BID vs. PBO 6 weeks	XZK BL 3.32, Wk 6 2.38; %CFB −34% *p* < 0.001 vs. BL PBO BL 3.35, Wk 6 3.26; *p* = NS vs. BL
Lin, 2005 [47]	Dyslipidemia *n* = 79	Primary	RYR 600 mg BID vs. PBO 8 weeks	%CFB, RYR −27.7%; PBO −1.5% *p* < 0.001 vs. BL and PBO
Becker, 2009 [48]	Dyslipidemia, statin intolerance *n* = 62	Primary	RYR 1800 mg BID (MK 3.06 mg BD) vs. PBO 24 weeks	%CFB Wk 24: RYR −21.3%; PBO −8.7% *p* = 0.011 vs. PBO for LDL-C concentrations at Wk 24
Bogsrud, 2010 [49]	Dyslipidemia, DM2 *n* = 42	NR	RYR 4 capsules/d (MK 7.2 mg/d) vs. PBO 16 weeks	%CFB, RYR vs. PBO: −23.0%; *p* < 0.001
Cicero, 2013 [50]	Mild dyslipidemia *n* = 25	Primary	MK 10 mg/d vs. PBO 4 weeks	%CFB, RYR vs. PBO: −22.0%; *p* < 0.01
Verhoeven, 2013 ^a^ [51]	Dyslipidemia *n* = 54	NR	RYR 2 capsules QD (MK 10 mg/d) vs. PBO 8 weeks	%CFB, RYR −22.2%; PBO +1.65%; *p* < 0.001
Moriarty, 2014 [52]	Dyslipidemia *n* = 116	Primary	XZK 600 mg BID or XZK 1200 mg BID vs. PBO 12 weeks	%CFB Wk 12: XZK 1200 mg −26.4%; *p* < 0.001 vs. BL and PBO %CFB Wk 12: XZK 2400 mg −27.0%; *p* < 0.001 vs. BL and PBO PBO +0.5% *p* = NS vs. BL
Heinz, 2016 [53]	Dyslipidemia *n* = 151	Primary	RYR 200 mg/d (MK 3 mg/d) vs. PBO 12 weeks	%CFB, RYR −14.8%; *p* < 0.001 vs. PBO %CFB, PBO −2.7%; *p* = NS vs. BL
Wang, 2019 [54]	Dyslipidemia *n* = 69	NR	GABA-rich RYR 250 mg capsules BID (RYR 335 mg/d; GABA 0.14 mg/d) MK-rich RYR 250 mg capsules BID (RYR 400 mg/d; MK 8 mg/d) vs. PBO 3 months	Median (mg/dL): RYR MK BL 153, 3 m 122; *p* < 0.05 vs. BL, RYR GABA, and PBO RYR GABA BL 151, 3 m 156; *p* = 0.009 vs. BL; *p* = NS vs. PBO PBO BL 154, 3 m 152; *p* = NS vs. BL
Minamizuka, 2021 [55]	Mild dyslipidemia *n* = 19	Primary	RYR 200 mg/d (MK 2 mg/d) + dietary therapy vs. dietary therapy alone 8 weeks	Median CFB: RYR −0.96; control −0.20; *p* = 0.030 vs. control
**Statin comparator**				
Xiaobin, 2007 [62]	CHD with dyslipidemia *n* = 130	Secondary	XZK (dose NA) vs. ATV (dose NA) 2 months	%CFB: XZK NA; ATV NA *p* < 0.01 for both vs. BL
Gheith, 2008 [61]	Nephrotic syndrome with dyslipidemia *n* = 72	NR	RYR 1.2 g/d for 1 month then 600 mg/d vs. FLV 20 mg/d or PBO 1 year	NR
Liu, 2011 [66]	Hyperlipidemia, carotid atherosclerosis *n* = 60	Secondary	LRRMP 350 mg/d vs. XZK 1.2 g/d vs. LOV 20 mg/d 6 months	NA *p* = NS intergroup comparison
Li, 2011 [65]	CHD and dyslipidemia *n* = 64	Secondary	XZK 1.2 g/d vs. LOV 40 mg/d 8 weeks	Lowered vs. BL *p* < 0.05 vs. BL; *p* = NS vs. LOV
Halbert, 2010 [63]	Dyslipidemia, statin intolerance *n* = 43	Primary and secondary	RYR 2400 mg BID (9.96 mg MK/d) vs. PRV 20 mg BID 12 weeks	%CFB: RYR −30.2%; PRV −27.0% ΔLDL-C (CFB RYR vs. PRV) ^a^: −10.7 mg/dL; *p* = NS
Ruscica, 2014 [59]	Dyslipidemia and metabolic syndrome *n* = 30	Primary	RYR 200 mg/d (Armolipid Plus^®^ [MK 3 mg/d]) vs. PRV 10 mg/d 8 weeks	%CFB: RYR −21.1%; PRV −22.6% both *p* < 0.0001 vs. BL; *p* = NS Armolipid Plus^®^ vs. PRV
Marazzi, 2017 [60]	CAD with PCI in preceding 12 months, HDS intolerant, poor response with LDS *n* = 100	Secondary	RYR 200 mg/d (Armolipid Plus^®^ [MK 3 mg/d]) plus LDS vs. LDS (ATV 5–10 mg/d, RSV 5 mg/d, or SMV 10–20 mg/d) 3 months	%CFB: RYR + LDS −26.8%; LDS −4.3% *p* < 0.0001 Armolipid Plus^®^ + LDS vs. LDS
Kou, 1997 [56]	Hyperlipidemia *n* = 108	Primary	XZK 1.2 g/d vs. SMV 10 mg/d 8 weeks	%CFB: XZK −28.0%; SMV −29.5% *p* < 0.001 for both vs. BL
Chen, 2002 [57]	Hypercholesterolemia *n* = 65	Primary	XZK 1.2 g/d vs. SMV 10 mg/d 4 weeks	%CFB: XZK −28.2%; SMV −22.7%; *p* = NA
Xue, 2017 [64]	Dyslipidemia *n* = 65	Primary	RYR 1.2 g/d vs. SMV 20 mg/d 4 weeks	%CFB: RYR −33.4%; SMV −30.9% *p* < 0.001 vs. BL for both *p* = NS for RYR vs. SMV
Cui, 2015 [58]	Unstable angina pectoris, statin intolerance ^b^ *n* = 90	Secondary	XZK 600 mg BID vs. SMV 20 mg QD vs. SMV stopped and restarted at 20 mg QD ^c^ 8 weeks	LDL-C (mg/dL): XZK BL 152, Wk 8 119; *p* < 0.05 vs. BL SMV BL 151, Wk 8 118; *p* < 0.05 vs. BL

Data are reported as mean values for baseline and at the end of the treatment period or mean change from baseline unless otherwise specified; ^a^ Obtained from linear regression models adjusted for baseline lipoprotein measure and body-mass index; ^b^ SMV-induced elevation of liver enzyme levels; ^c^ Subjects restarted SMV 20 mg QD once their liver enzyme levels had returned to normal. ATV = atorvastatin; BID = twice daily; BL = baseline; CAD = coronary artery disease; CFB = change from baseline; CHD = coronary heart disease; DM2 = type 2 diabetes mellitus; FLV = fluvastatin; GABA = gamma-aminobutyric acid; HDS = high-dose statin; LDS = low-dose statin; LOV = lovastatin; LRRMP = lipid-reducing red rice minute powder; MK = monacolin K; NA = not available; NR = not reported; NS = not significant; PBO = placebo; PCI = percutaneous coronary intervention; PRV = pravastatin; RSV = rosuvastatin; QD = once daily; RYR = red yeast rice; SMV = simvastatin; Wk = week; XZK = Xuezhikang.

**Table 4:** Summary of effects of RYR on CVD risks.

Study	Population	Interventions	Incidence of CV Outcomes						
			Revasculariz-Ation	Fatal MI	Non-Fatal MI	Stroke	CV Events ^a^	CV Mortality	All-Cause Mortality
**Clinical trials**									
Cui, 2015 [58]	Unstable angina pectoris, statin intolerance *n* = 90	XZK 600 mg BID vs. SMV 20 mg QD vs. SMV stopped and restarted at 20 mg QD Duration = 8 weeks	–	–	–	–	XZK 3.3% SMV 3.3% SMV stopped 13.3% (*p* < 0.05 for SMV stopped vs. XZK and SMV)	–	–
Lu, 2008 [69]	Previous MI, average LDL-C levels *n* = ~5000	XZK 600 mg BID vs. PBO Duration = 4.5 years	33% reduction	–	–	–	XZK 5.7% PBO 10.4% ^b^ 45% reduction	30% reduction	33% reduction
Li, 2010 [70]	Subgroup: previous MI, hypertensive *n* = 2704	XZK 600 mg BID vs. PBO Duration = 4.5 years	–	XZK 1.0% PBO 1.3% (*p* = NS) 29% reduction	XZK 2.2% PBO 5.4% (*p* < 0.001) 60% reduction	XZK 3.5% PBO 5.1% (*p* = 0.06) 32% reduction	XZK 6.7% PBO 11.9% (*p* = 0.0214) 43% reduction	XZK 4.5% PBO 6.5% (*p* = 0.001) 30% reduction	XZK 5.9% PBO 9.3% (*p* = 0.001) 36% reduction
Li, 2009 [71]	Subgroup: previous MI, elderly, hypertensive, average LDL-C levels *n* = 1530	XZK 600 mg BID vs. PBO Duration = 4.5 years	–	–	–		XZK 8.8% PBO 14.3% 38% reduction	XZK 6.4% PBO 9.0% 29% reduction	
**Meta-analyses**									
Sungthong, 2020 [72]	Previous MI, borderline hypercholesterolemia *n* = 10,699	RYR 600 mg BID vs. PBO Duration = 4 weeks to 4.5 years	RR 0.58 (95% CI 0.48, 0.71) *p* < 0.00001 42% reduction	RR 0.78 (95% CI 0.55, 1.10) *p* = 0.16 22% reduction	RR 0.42 (95% CI 0.34, 0.52) *p* < 0.00001 58% reduction	–	–	–	–
Yuan, 2022 [73]	Metabolic syndrome *n* = 5440	XZK vs. control (PBO or routine treatment) Duration = 4 weeks to 4.5 years	–	–	–	–	MACE: RR 0.54 (95% CI 0.43, 0.66); *p* < 0.00001 46% reduction	–	RR 0.62 (95% CI 0.49, 0.78); *p* < 0.0001 38% reduction
**Real-world retrospective cohort study**									
Chang, 2022 [74]	No history of stroke *n* = 69,446	RYR vs. LOV Duration = NR	–	–	–	– RYR 3.97/1000 PYs LOV 6.99/100 PYs 35% reduction	–	–	–

^a^ Includes non-fatal MI, fatal MI, and death; ^b^ Includes non-fatal MI and deaths from CHD. BID = twice daily; CHD = coronary heart disease; CI = confidence interval; CV = cardiovascular; CVD = cardiovascular disease; LDL-C = low-density lipoprotein cholesterol; LOV = lovastatin; MACE = major adverse cardiac events; MI = myocardial infarction; NR = not reported; NS = not significant; PBO = placebo; PY = person-year; QD = once daily; RR = risk ratio; RYR = red yeast rice; SMV = simvastatin; XZK = Xuezhikang.

**Table 5:** Summary of AEs reported in key randomized controlled trials of RYR in subjects with dyslipidemia.

Study	Number of Subjects			Type of AE	*n* (%)		
	N (RYR Dose; MK [Lovastatin] Dose)		Control		RYR		Control
**Placebo comparator**							
Heber, 1999 [44]	42 (2.4 g/d; 4.8 mg/d)		41	Serious Total	0 (0) 1 (2)		0 (0) 3 (7)
				Musculoskeletal chest pain	1 (2)		0 (0)
				Headache	0 (0)		1 (2)
				Pneumonia	0 (0)		1 (2)
				Rash/pruritus/skin allergy	0 (0)		1 (2)
Lin, 2005 [47]	37 (1.2 g/d; 11.4 mg/d)		38	Serious Breast carcinoma (not related to RYR) Total	1 (3) 1 (3) 21 (57)		0 (0) 28 (74)
				Increased CPK	1 (3)		0 (0)
				Increased ALT	1 (3)		0 (0)
				Diarrhea	0 (0)		1 (3)
				Nausea	0 (0)		1 (3)
				Leukopenia	0 (0)		1 (3)
				LDH increase	1 (3)		0 (0)
Becker, 2009 [48] ^a^	31 (3.6 g/d; 6 mg/d)		31	Total	NR		NR
				Serious	NR		NR
				Myalgia	2 (6)		1 (3)
				Loose stools	1 (3)		0 (0)
				Dizziness	1 (3)		0 (0)
Bogsrud, 2010 [49]	22 (4 capsules; 7.2 mg/d)		20	Serious Total	0 (0) 8 (36)		0 (0) 1 (5)
				Back pain	1 (5)		0 (0)
				Increased CPK	1 (5)		0 (0)
				Diarrhea	2 (9)		0 (0)
				Flatulence	1 (5)		1 (5)
				Crohn’s disease	1 (5)		0 (0)
				General discomfort	1 (5)		0 (0)
				Influenza	1 (5)		0 (0)
Verhoeven, 2013 [51]	31 (2 capsules/day; 10.05 mg/d)		21	Serious Total	NR NR		NR NR
				Muscle stiffness	2 (6)		0 (0)
				Muscle cramps	3 (10)		1 (5)
				Myalgia	4 (13)		2 (10)
				Increased CPK	5 (16)		3 (14)
				Liver pain	0 (0)		1 (5)
				Belches	1 (3)		0 (0)
				Erectile dysfunction	0 (0)		1 (5)
				Insomnia	1 (3)		1 (5)
				Pruritus	0 (0)		1 (5)
Moriarty, 2014 [52] ^b^	XZK (1.2 g/d) *n* = 36	XZK (2.4 g/d) *n* = 42	37	Serious Fracture, extremity (not related to RYR) Pulmonary embolism (not related to RYR) Thyroid cancer (not related to RYR) Total	1.2 g/d 0 (0) 0 (0) 1 (3) 17 (47)	2.4 g/d 1 (2) 1 (2) 0 (0) 22 (52)	PBO 0 (0) 0 (0) 0 (0) 19 (51)
				Musculoskeletal/connective tissue disorders	4 (11)	5 (12)	1 (3)
				- Muscle spasm	0 (0)	2 (5)	0 (0)
				- Myalgia	1 (3)	2 (5)	0 (0)
				- Jaw pain	1 (3)	0 (0)	0 (0)
				Investigations/laboratory abnormalities	2 (6)	1 (2)	6 (16)
				- Increased CPK	0 (0)	0 (0)	2 (5)
				- Increased ALT	0 (0)	0 (0)	2 (5)
				- Increased AST - Increased leukocyte count	0 (0) 2 (6)	0 (0) 0 (0)	2 (5) 0 (0)
				Gastrointestinal disorders	5 (14)	10 (24)	10 (27)
				- Diarrhea	2 (6)	0 (0)	1 (3)
				- Dyspepsia	3 (8)	1 (2)	1 (3)
				- Nausea	0 (0)	2 (5)	2 (5)
				- Abdominal discomfort	0 (0)	0 (0)	2 (5)
				- Epigastric pain	1 (3)	0 (0)	0 (0)
				Nervous system disorders	1 (3)	3 (7)	3 (8)
				- Headache	1 (3)	2 (5)	2 (5)
				Infections	4 (11)	5 (12)	4 (11)
				- URTI	0 (0)	2 (5)	3 (8)
				Rash	1 (3)	0 (0)	0 (0)
				Skin flushing	0 (0)	0 (0)	1 (3)
Wang, 2019 [54] ^c^	MK-RYR (400 mg/d; 8 mg/d) *n* = 23	GABA-RYR (335 mg/d; NA) *n* = 23	23	Serious Total Elevated creatinine Increased ALT Increased AST	0 (0) 1 (4) 0 (0) 1 (4) 1 (4)	0 (0) 1 (4) 0 (0) 1 (4) 1 (4)	0 (0) 3 (13) 1 (4) 0 (0) 0 (0)
				Poor general health	0 (0)	0 (0)	1 (4)
				Anxiety	1 (4)	0 (0)	0 (0)
				Skin allergy	0 (0)	0 (0)	1 (4)
Minamizuka, 2021 [55]	10 (200 mg/d; 2 mg/d)		8	Serious	NR		NR
				Skin rash	0 (0)		0 (0)
				Muscle pain	0 (0)		0 (0)
				Total	0 (0)		0 (0)
**Statin comparator**							
Xiaobin, 2007 [62]	XZK; NA		ATV; NA *n* = 130 overall		NA		NA
Gheith, 2008 [61]	RYR 1.2 g/d for 1 month then 600 mg/d *n* = 20		FLV 20 mg/d *n* = 30 PBO *n* = 22		NR		NR
Liu, 2011 [66]	LRRMP (350 mg/d; NA) *n* = 20; XZK (1.2 g/d; NA) *n* = 20		LOV 20 mg/d *n* = 20	Serious Total	0 (0) NA		1 (5) ^d^ NA
Li, 2011 [65]	XZK (1.2 g/d; NA) *n* = 32		LOV 40 mg/d *n* = 32		NA		NA
Halbert, 2010 [63]	RYR (4.8 g/d; 9.96 mg/d) *n* = 21		PRV 40 mg/d *n* = 22	Serious Total	NR NR		NR NR
				Persistent myalgia only			
				- Generalized	0 (0)		3 (14)
				- Local	2 (10)		1 (5)
				- Local and generalized	2 (10)		4 (18)
				Persistent and intermittent myalgia			
				- Generalized	1 (5)		6 (27)
				- Local	4 (19)		3 (14)
				- Local and generalized	5 (24)		8 (36)
				Muscle weakness	1 (5)		1 (5)
				Abdominal gas, bloating	2 (10)		0 (0)
				Alopecia	2 (10)		0 (0)
				Arthralgia	1 (5)		1 (5)
				Back pain	5 (24)		6 (27)
				Diarrhea	2 (10)		0 (0)
				Dizziness	0 (0)		2 (9)
				Dyspepsia	1 (5)		0
				Fatigue	0		3 (14)
				Fracture, extremity	1 (5)		0
				Headache	2 (10)		2 (9)
				Motor co-ordination decreased, left hand	0		1 (5)
Ruscica, 2014 [59]	RYR (200 mg/d; 3 mg/d) *n* = 30		PRV 10 mg/d *n* = 30	Serious Total	NR NR		NR NR
Marazzi, 2017 [60]	RYR (200 mg/d; 3 mg/d) *n* = 50		SMV 10–20 mg/d or ATV 5–10 mg/d or RSV 5 mg/d *n* = 50	Serious	NR		NR
				Total	NR		NR
				Musculoskeletal discomfort	3 (6)		3 (6)
				Hepatobiliary disorders	0 (0)		0 (0)
				Gastrointestinal disorders	2 (4)		1 (2)
				Metabolic disorders	0 (0)		0 (0)
Kou, 1997 [56]	XZK (1.2 g/d; NA) *n* = 53		SMV 10 mg/d *n* = 55	Severe Total	NA NA		NA NA
Chen, 2002 [57]	XZK (1.2 g/d; NA) *n* = NA ^e^		SMV 10 mg/d *n* = NA ^e^	Serious Total	0 (0) 0 (0)		0 (0) 0 (0)
Xue, 2017 [64]	RYR (1.2 g/d; NA) *n* = 27		SMV 20 mg/d *n* = 33	Serious Total	0 (0) 0 (0)		0 (0) 0 (0)
Cui, 2015 [58]	XZK 600 mg BID *n* = 30		SMV 20 mg QD *n* = 30 SMV stopped and restarted at 20 mg QD *n* = 30		NR		NR

^a^ AEs leading to study discontinuation; ^b^ AEs leading to discontinuations and/or AEs reported in ≥2 subjects; ^c^ Report described AEs leading to discontinuation; ^d^ One patient dropped out due to transaminase elevation; listed as SAE here; ^e^ Total number of patients in study = 65. AE = adverse event; ALT = alanine aminotransferase; AST = aspartate aminotransferase; ATV = atorvastatin; BID = twice daily; CPK = creatine phosphokinase; FLV = fluvastatin; GABA = gamma-aminobutyric acid-rich RYR; LDH = lactate dehydrogenase; LRRMP = lipid-reducing red rice minute powder; LOV = lovastatin; MK = monacolin K; NA = not available; NR = not reported; QD = once daily; PRV = pravastatin; RSV = rosuvastatin; RYR = red yeast rice; SAE = serious adverse event; SMV = simvastatin; URTI = upper respiratory tract infection; XZK = Xuezhikang.

## Data Availability

Data sharing is not applicable to this article as no datasets were generated or analyzed during the current study.

## Supplementary Materials

The following supporting information can be downloaded at: https://www.mdpi.com/article/10.3390/nu15102288/s1, Table S1. Other components of monacolin K formulation in key randomized controlled trials of RYR versus placebo or statins in subjects with dyslipidemia; Table S2. Summary of serum lipid outcomes in key randomized controlled trials of RYR versus placebo or statins in subjects with dyslipidemia; Table S3. Summary of RYR safety reported by meta-analyses; Table S4. Summary of RYR adverse drug reaction reports collected by surveillance systems.
