# Supplementary material for: Red Yeast Rice for the Improvement of Lipid Profiles in Mild-to-Moderate Hypercholesterolemia: A Narrative Review
Source: Nutrients. 2023 May 12;15(10):2288. doi: 10.3390/nu15102288 (PMC10221652; doi:10.3390/nu15102288)
Supplement: Supplementary file 1 [file nutrients-15-02288-s001.zip › nutrients-2372228-supplementary.pdf]

nutrients MDPI

Supplementary Materials:

Supplemental Table S1. Other components of monacolin K formulation in key randomized controlled trials of RYR versus placebo or statins in subjects with dyslipidemia.

Study	Other Components
**Placebo comparator**	
Heber, 1999 [44]	Rice starch, fiber, protein, moisture, natural pigment, ash, organic phosphorus, trace elements, dihydromonacolin, monacolin I, monacolin II (hydroxy-acid form), monacolin III, monacolin IV, monacolin V, monacolin VI, saturated (palmitic and stearic) fatty acids, mono- and polyunsaturated fatty acids
Zhao, 2003 [45] and Zhao, 2004 [46]	Eight other monacolins, unsaturated fatty acids, sterols, isoflavones, glycerides, trace elements, other substances
Lin, 2005 [47]	Protein, starch, fat (linoleic acid, oleic acid, palmitic acid, stearic acid, ergosterol), fiber, water, other statins, gamma-aminobutyric acid, alkaloids, glycosides, flavonoids, natural pigments, ethanol extracts, water extracts, citrinin, trace elements
Becker, 2009 [48]	Monacolin JA, monacolin J, monacolin XA, monacolin KA, monacolin LA, monacolin L, monacolin M, dihydromonacolin K, citrinin
Bogsrud, 2010 [49]	Other monacolins
Cicero, 2013 [50]	Highly purified, without detectable dehydromonacolins, decalin derivatives, contaminants
Verhoeven, 2013 [51]	Ubiquinone (co-enzyme Q-10), procyanidins, lecithin
Moriarty, 2014 [52]	Other monacolins, phytosterols
Heinz, 2016 [53]	Coenzyme Q10, astaxanthin, folic acid
Wang, 2019 [54]	Other monacolins, gamma-aminobutyric acid, pigments, dimerumic acid, citrinin
Minamizuka, 2021 [55]	Other monacolins, pigments, organic acids (including gamma-aminobutyric acid), amino acids
**Statin comparator**	
Halbert, 2010 [63]	Other monacolins, citrinin, trace metals
Ruscica, 2014 [59]	Berberine, policosanol, astaxanthin, coenzyme Q10, folic acid
Marazzi, 2017 [60]	Berberine, policosanol, astaxanthin, coenzyme Q10, folic acid
Cui, 2015 [58]	Other monacolins, unsaturated fatty acids, sterols, alkaloids, essential amino acids, flavonoids, trace metals

RYR = red yeast rice.

Supplemental Table S2. Summary of serum lipid outcomes in key randomized controlled trials of RYR versus placebo or statins in subjects with dyslipidemia.

Study	LDL-C (mmol/L)	TC (mmol/L)	HDL-C (mmol/L)	TG (mmol/L)	ApoA-1 (mg/L)	ApoB (mg/L)
**Placebo comparator**						
Heber, 1999 [44]	CFB:<br>RYR –1.01;<br>PBO –0.13<br>$p < 0.001$ vs. PBO	CFB:<br>RYR –1.03;<br>PBO –0.13<br>$p < 0.05$ vs. PBO	RYR BL 1.29, Wk 12 1.29<br>PBO BL 1.19, Wk 12 1.19<br>p = NS vs. PBO	CFB:<br>RYR –0.10;<br>PBO +0.03<br>$p = 0.05$ vs. PBO	NR	NR
Zhao, 2003 [45];<br>Zhao, 2004 [46]	XZK BL 3.32, Wk 6 2.38; %CFB −34% $p < 0.001$ vs. BL<br>PBO BL 3.35, Wk 6 3.26; p = NS vs. BL	XZK BL 5.37, Wk 6 4.36; %CFB −20% $p < 0.001$<br>PBO BL 5.37, Wk 6 5.30; p = NS	XZK BL 1.15, Wk 6 1.35; %CFB +18% $p < 0.001$<br>PBO BL 1.15, Wk 6 1.15; p = NS	XZK BL 1.77, Wk 6 1.22; %CFB −32% $p < 0.001$<br>PBO BL 1.74, Wk 6 1.68; p = NS	%CFB +13%; $p < 0.001$	%CFB −27%; $p < 0.001$
Lin, 2005 [47]	%CFB:<br>RYR −27.7%; $p < 0.001$ vs. BL and PBO<br>PBO −1.5%	%CFB:<br>RYR −21.5%; $p < 0.001$ vs. BL and PBO<br>PBO −0.4%	%CFB:<br>RYR +0.9%; p = NS vs. BL and PBO<br>PBO +1.0%	%CFB:<br>RYR −15.8%; $p < 0.05$ vs. BL and PBO<br>PBO +1.0%	%CFB:<br>RYR +3.4%; p = NS vs. BL and PBO<br>PBO +2.3%	%CFB:<br>RYR −26.0%; $p < 0.001$ vs. BL and PBO<br>PBO −3.9%
Becker, 2009 [48]	%CFB:<br>RYR −21.3%;<br>PBO −8.7%<br>$p = 0.011$	%CFB:<br>RYR −14.9%;<br>PBO −5.3%<br>$p = 0.016$	%CFB:<br>RYR +8.6%; PBO +7.9%<br>p = NS	%CFB:<br>RYR −7.2%;<br>PBO −1.4%<br>p = NS	NR	NR
Bogsrud, 2010 [49]	%CFB RYR vs. PBO:<br>−23.0%<br>$p < 0.001$	%CFB RYR vs. PBO:<br>−15.5%<br>$p < 0.001$	RYR BL 1.62, Wk 16 1.71<br>PBO BL 1.35, Wk 16 1.48<br>p = NS RYR vs. PBO	RYR BL 1.01, Wk 16 0.90<br>PBO BL 1.29, Wk 16 1.51<br>p = NS RYR vs. PBO	RYR BL 1.46, Wk 16 1.61<br>PBO BL 1.35, Wk 16 1.47<br>p = NS RYR vs. PBO	RYR BL 0.99, Wk 16 0.77<br>PBO BL 1.11, Wk 16 1.11<br>$p < 0.001$ RYR vs. PBO
Cicero, 2013 [50]	%CFB RYR vs. PBO:<br>−22.0%; $p < 0.01$	%CFB RYR vs. PBO:<br>−12.5%; $p < 0.01$	%CFB RYR vs. PBO: NS	%CFB RYR vs. PBO: NS	NR	NR
Verhoeven, 2013 [51]	%CFB:<br>RYR −22.2%; PBO +1.65%<br>$p < 0.001$	%CFB:<br>RYR −14.6%; PBO +1.2%<br>$p < 0.001$	%CFB:<br>RYR +3.0; PBO −0.3%<br>p = NS	%CFB:<br>RYR −13.8%; PBO +5.0%<br>$p = 0.05$	NR	NR
Moriarty, 2014 [52]	%CFB:<br>RYR 1200 mg	LS mean %CFB:<br>RYR 1200 mg −17.8%; $p <$	LS mean %CFB:<br>RYR 1200 mg +4.3%; $p =$	LS mean %CFB:<br>RYR 1200 mg	LS mean %CFB:<br>RYR 1200 mg +5.8%; $p <$	LS mean %CFB:<br>RYR 1200 mg −19.0%; $p <$

	−26.4%; $p < 0.001$ vs. BL and PBO<br>RYR 2400 mg −27.0%; $p <$ 0.001 vs. BL and PBO<br>PBO +0.5%, p = NS vs. BL	0.001 vs. PBO<br>RYR 2400 mg −18.5%; $p <$ 0.001 vs. PBO<br>PBO +0.4%	NS vs. PBO<br>RYR 2400 mg +5.2%; p = NS vs. PBO<br>PBO −2.2%	−8.0%; p = NS vs. PBO<br>RYR 2400 mg −5.9%; p = NS vs. PBO<br>PBO +12.0%	0.001 vs. PBO<br>RYR 2400 mg +3.9%, p = NS vs. PBO<br>PBO 0%	0.001 vs. PBO<br>RYR 2400 mg −21.2%; $p <$ 0.001 vs. PBO<br>PBO +2.9%
Heinz, 2016 [53]	%CFB:<br>RYR −14.8%; $p < 0.001$ vs. PBO<br>PBO −2.7%; p = NS vs. BL	%CFB:<br>RYR −11.2%; $p < 0.001$ vs. PBO<br>PBO −1.0%; p = NS vs. BL	%CFB:<br>RYR +0.7%; p = NS vs. PBO<br>PBO +0.2%; p = NS vs. BL	%CFB:<br>RYR −5.0%; $p < 0.01$ vs. BL; p = NS vs. PBO<br>PBO −0.4%	NR	NR
Wang, 2019 [54]	Median (mg/dL):<br>RYR MK BL 153, 3 m 122; $p < 0.05$ vs. BL, RYR GABA, and PBO<br>RYR GABA BL 151, 3 m 156; $p = 0.009$ vs. BL; p = NS vs. PBO<br>PBO BL 154, 3 m 152; p = NS vs. BL	Median (mg/dL):<br>RYR MK BL 237, 3 m 192.5; $p < 0.001$ vs. BL, $p < 0.05$ vs. RYR GABA and PBO<br>RYR GABA BL 235, 3 m 237; p = NS vs. BL and PBO<br>PBO BL 230, 3 m 234; p = NS vs. BL	Median (mg/dL):<br>RYR MK BL 51, 3 m 54; p = NS vs. BL, RYR GABA, and PBO<br>RYR GABA BL 56, 3 m 52; p = NS vs. BL and PBO<br>PBO BL 50, 3 m 49; p = NS vs. BL	Median (mg/dL):<br>RYR MK BL 133, 3 m 113; p = NS vs. BL, RYR GABA, and PBO<br>RYR GABA BL 112, 3 m 104; p = NS vs. BL and PBO<br>PBO BL 148, 3 m 161; p = NS vs. BL	NR	NR
Minamizuka, 2021 [55]	Median CFB:<br>RYR −0.96; control −0.20<br>$p = 0.030$	Median CFB:<br>RYR −0.92; control 0.00<br>$p = 0.014$	Median CFB:<br>RYR −0.13; control 0.03<br>$p = 0.082$	Median CFB:<br>RYR 0.24; control −0.05<br>p = NS	NR	Median CFB (g/L):<br>RYR −0.18; control 0.03<br>$p = 0.011$
**Statin comparator**						
Xiaobin, 2007 [62]	%CFB: XZK NA; ATV NA<br>$p < 0.01$ vs. BL for each; p = NA XZK vs. ATV	%CFB: XZK NA; ATV NA<br>$p < 0.05$ vs. BL for each; p = NA XZK vs. ATV	%CFB: XZK NA; ATV NA<br>$p < 0.05$ vs. BL for each; p = NA XZK vs. ATV	%CFB: XZK NA; ATV NA<br>$p < 0.01$ vs. BL for each; p = NA XZK vs. ATV	NR	NR
Gheith, 2008 [61]	NR	Mean (mg/dL)<br>XZK BL 457, 1 yr 303; FLV BL 436, 1 yr 302; PBO BL 463, 1 yr 348; $p = 0.003$ for FLV vs. PBO	NR	NR	NR	NR

Liu, 2011 [66]	%CFB: significantly lowered for all groups; intergroup comparisons (XZK, LRRMP, LOV) p = NS	%CFB: significantly lowered for all groups; intergroup comparisons (XZK, LRRMP, LOV) p = NS	%CFB: not significantly lowered for all groups	%CFB: significantly lowered for all groups; intergroup comparisons (XZK, LRRMP, LOV) p = NS	NR	NR
Li, 2011 [65]	Reduced vs. control in both groups $p = 0.05$	Reduced vs. control in both groups $p = 0.05$	NR	Reduced vs. control in both groups $p = 0.05$	NR	NR
Halbert, 2010 [63]	%CFB:<br>RYR −30.2%; PRV −27.0%<br>ΔLDL-C (CFB RYR vs. PRV): −10.7 mg/dL; p = NS	%CFB:<br>RYR −23.0%; PRV −19.6%<br>ΔTC (CFB RYR vs. PRV): −9.6 mg/dL; p = NS	%CFB:<br>RYR −3.8%; PRV +0.2%<br>ΔHDL-C (CFB RYR vs. PRV):<br>−2.5 mg/dL; p = NS	%CFB:<br>RYR −7.8%; PRV −7.0%<br>ΔTG (CFB RYR vs. PRV): 0.5 mg/dL; p = NS	NR	NR
Ruscica, 2014 [59]	Armolipid Plus® BL 3.91, Wk 8 3.09<br>PRV BL 3.97, Wk 8 3.07<br>$p < 0.0001$ for both vs. BL<br>p = NS Armolipid Plus® vs. PRV	Armolipid Plus® BL 6.2, Wk 8 5.4<br>PRV BL 6.41, Wk 8 5.38<br>$p < 0.0001$ for both vs. BL<br>p =NS Armolipid Plus® vs. PRV	Armolipid Plus® BL 1.04, Wk 8 1.09<br>PRV BL 1.10, Wk 8 1.11<br>p = NS PRV vs. BL<br>$p < 0.05$ Armolipid Plus® vs. BL<br>p = NS Armolipid Plus® vs. PRV	Armolipid Plus® BL 2.44, Wk 8 2.21<br>PRV BL 2.55, Wk 8 2.43<br>p = NS for both vs. BL<br>p = NS Armolipid Plus® vs. PRV	NR	NR
Marazzi, 2017 [60]	%CFB: RYR + LDS −26.8%; LDS −4.3%<br>$p <0.0001$ for Armolipid Plus® + LDS vs. LDS	%CFB: RYR + LDS −17.5%; LDS −3.5%<br>$p < 0.0001$ for Armolipid Plus® + LDS vs. LDS	%CFB: RYR + LDS +8.8%; LDS +3.7%<br>$p = 0.02$ for Armolipid Plus® + LDS vs. LDS	%CFB: RYR + LDS −10.2%; LDS −7.9%<br>p = NS for Armolipid Plus® + LDS vs. LDS	NR	NR
Kou, 1997 [56]	%CFB: XZK −28.0%; SMV −29.5%<br>p = NS XZK vs. SMV	%CFB: XZK −23.0%; SMV −23.3%<br>p = NS XZK vs. SMV	%CFB: XZK +5.0%; SMV +14.3%<br>p = NS XZK vs. SMV	%CFB: XZK −28.1%; SMV −29.5%<br>p = NS XZK vs. SMV	NR	NR

Chen, 2002 [57]	%CFB: XZK −28.2%; SMV −22.7%<br>p = NS XZK vs. SMV	%CFB: XZK −21.8%; SMV −21.3%<br>p = NS XZK vs. SMV	%CFB: XZK +6.2%; SMV +5.7%<br>p = NS XZK vs. SMV	%CFB: XZK −18.1%; SMV −1.6%<br>$p < 0.001$ XZK vs. SMV	NR	NR
Xue, 2017 [64]	%CFB: RYR −33.4%; SMV −30.9%<br>$p < 0.001$ for both vs. BL<br>p = NS RYR vs. SMV	%CFB: RYR −18.5%; SMV −19.6%<br>$p < 0.001$ for both vs. BL<br>p = NS RYR vs. SMV	%CFB:<br>p = NS for both vs. BL	%CFB:<br>p = NS for both vs. BL	NR	NR
Cui, 2015 [58]	LDL-C (mg/dL):<br>XZK BL 152, Wk 8 119; p <0.05 vs. BL<br>SMV BL 151, Wk 8 118; p < 0.05 vs. BL	TC (mg/dL):<br>XZK BL 200, Wk 8 170; p < 0.05 vs. BL<br>SMV BL 201, Wk 8 156; p <0.05 vs. BL	HDL-C (mg/dL):<br>XZK BL 41, Wk 8 49; p < 0.05 vs. BL and SMV<br>SMV BL 42, Wk 8 44; p = NS vs. BL	TG (mg/dL):<br>XZK BL 189, Wk 8 146; p < 0.05 vs. BL and SMV<br>SMV BL 191, Wk 8 168; p < 0.05 vs. BL	NR	NR

Apo = apolipoprotein; ATV = atorvastatin; BL = baseline; CFB = change from baseline; FLV = fluvastatin; GABA = gamma-aminobutyric acid; HDL-C = high-density lipoprotein cholesterol; HDS = high-dose statin; LDL-C = low-density lipoprotein cholesterol; LDS = low-dose statin (ATV 5–10 mg/d, RSV 5 mg/d, or SMV 10–20 mg/d); LOV = lovastatin; LRRMP = lipid-reducing red rice minute powder; LS = least squares; MK = monacolin K; NA = not available; NR = not reported; NS = not significant; PBO = placebo; PRV = pravastatin; QD = once daily; RYR = red yeast rice; SMV = simvastatin; TC = total cholesterol; TG = triglycerides; Wk = week; XZK = Xuezhikang.

Supplemental Table S**3.** Summary of RYR safety reported by meta-analyses.

Authors	Study Dates	Number of Studies	RYR Dosage[a]	Comparators	Principal AE Findings
Gerards et al. [10]	Up to November 2014	20	RYR 1200–4800 mg/d (MK 4.8–24 mg/d)	Inactive control (13); statin (3); non-statin active control (4)	Incidence of liver abnormalities and kidney injury: 0–5%: did not differ between RYR and control<br>Incidence of muscle symptoms: 0–23.8% with RYR, 0–36% with controls<br>Rhabdomyolysis: not observed
Li et al. [11]	Up to September 2021	15	RYR or XZK 200–2400 mg/d	Placebo (9); statins (3); phytosterols (1); nattokinase (1); nutraceuticals (1)	Incidence of AEs with RYR was similar to control (RYR alone: RR 1.18; 95% CI 0.91 to 1.54; $p = 0.21$. RYR combination: RR 1.63; 95% CI 0.22 to 11.83; $p = 0.63$)
Fogacci et al. [86][b]	Up to 2019	53	RYR 100–4800 mg/d	Placebo (47); statins (6); non-statin active control (2)	RYR was not associated with musculoskeletal disorders (OR=0.94, 95% CI 0.53 to 1.65)<br>Risk of non-musculoskeletal disorders reduced (OR=0.59, 95% CI 0.50 to 0.69)<br>Risk of serious AEs reduced (OR=0.54, 95% CI 0.46 to 0.64)<br>Results were consistent across subgroups
Shang et al. [95]	Up to September 2011	22[c]	XZK 600–1800 mg/d	Conventional therapy (11); statin + conventional therapy (6); statin (4)	Most commonly reported AEs: intestinal disturbances, dizziness, high serum alanine aminotransferase, high serum creatine kinase, high serum creatinine, high blood urea nitrogen, and skin itch<br>AEs were not significantly different between XZK and control
Cicero et al. [76]	Up to February 2021	12	Armolipid Plus®	Placebo (11); low-dose statin (1)	Armolipid Plus® produced a slight but clinically insignificant increase in serum ALT without affecting AST or CPK<br>Armolipid Plus® was not associated with increased risks of musculoskeletal disorders or gastrointestinal disorders

[a]Some studies included combinations of RYR with nutraceuticals or a statin; [b]Two studies had placebo and a statin as control; [c]Studies in patients with coronary heart disease.

AE = adverse event; ALT = alanine aminotransferase; AST = aspartate aminotransferase; CI = confidence interval; CPK = creatine phosphokinase; OR = odds ratio; RR = relative risk; RYR = red yeast rice; XZK = Xuezhikang.

Supplemental Table S4. Summary of RYR adverse drug reaction reports collected by surveillance systems.

**Surveillance System**	**Collection Period**	**Total Number of Case Reports, *n***	**Total Number of ADRs, *n***	**Causality of ADRs, *n***	**Most Frequently Reported Adverse Drug Reactions by SOC, *n* (%)**					
					**Musculo-skeletal**	**Gastro-intestinal**	**Hepato-biliary**	**Skin and Subcutaneous**	**General**	**Nervous System**
Italian Surveillance System of Natural Health Products [96]	2002–2015	52	55	Certain 1; probable 31; possible 18; unlikely 3; unassessable 2	20 (36)	12 (22)	10 (18)	9 (16)	–	2 (4)
Netherlands Pharmacovigilance Centre Lareb [97]	2007–2020	94	187	Certain 2; probable/likely 24; possible 61; unlikely 7	64 (34)	33 (18)	3 (2)	6 (3)	23 (12)	16 (9)
Post-marketing product-based (Armolipid®/Armolipid Plus®) database [75]	2004–2019	542	855	–	148 (17)	293 (34)	26 (3)	–	–	–

ADRs = adverse drug reactions; RYR = red yeast rice; SOC = system organ class.
